# Trophic Transfer without Biomagnification of Cadmium in a Soybean-Dodder Parasitic System

**DOI:** 10.3390/plants10122690

**Published:** 2021-12-07

**Authors:** Bin J. W. Chen, Jing Xu, Xinyu Wang

**Affiliations:** College of Biology and the Environment, Nanjing Forestry University, Nanjing 210037, China; xujing0323@outlook.com (J.X.); Wangxinyu1007@outlook.com (X.W.)

**Keywords:** *Cuscuta*, food chain, feeding mode, heavy metal, holoparasite, host, parasitic plants

## Abstract

Cadmium (Cd) is among the most available and most toxic heavy metals taken up by plants from soil. Compared to the classic plant-animal food chains, the host-parasitic plant food chains have, thus far, been largely overlooked in the studies of Cd trophic transfer. To investigate the pattern of Cd transfer during the infection of parasitic plants on Cd-contaminated hosts, we conducted a controlled experiment that grew soybeans parasitized by Chinese dodders (*Cuscuta chinensis*) in soil with different levels of Cd treatment, and examined the concentration, accumulation, allocation and transfer coefficients of Cd within this parasitic system. Results showed that among all components, dodders accounted for more than 40% biomass of the whole system but had the lowest Cd concentration and accumulated the least amount of Cd. The transfer coefficient of Cd between soybean stems and dodders was much lower than 1, and was also significantly lower than that between soybean stems and soybean leaves. All these features were continuously strengthened with the increase of Cd treatment levels. The results suggested no evidence of Cd biomagnification in dodders parasitizing Cd-contaminated hosts, and implied that the Cd transfer from hosts to dodders may be a selective process.

## 1. Introduction

Along with worldwide industrialization over the last century, environmental pollution has become an important global issue [1]. Heavy metals, i.e., (semi-)metallic elements with an atomic density > 5 g·cm^−3^ [2], have been considered as one of the major types of pollutants [3,4]. Nowadays, the soil has been heavily contaminated by heavy metals, generating serious threats to food safety [5,6] and human health [2,7]. Among various heavy metals, cadmium (Cd) is a non-essential element and can be naturally found in the earth’s crust at low concentrations [7]. In addition to some natural processes (e.g., rock weathering and volcanic eruptions), the major sources of Cd contamination in topsoil originate from anthropogenic activities, including phosphate fertilizer applications, industrial waste disposal, fossil fuel combustions, and sewage sludge amendments [4,8,9]. Owing to its relative mobility in soil, Cd is among the most available heavy metals for plant uptake [6,8]. Meanwhile, due to chemical similarities to the divalent ions of some essential metals (e.g., zinc, iron and calcium), Cd ions in soil can easily enter root cells via less-specialized transporters and channels of those ions on the plasma membranes [10]. Cd is also among the most toxic metals to plants [11].

The transfer and accumulation of Cd through food chains have received great attention for more than half a century [9]. There is ample evidence showing that Cd can be biomagnified (i.e., more concentrated) through the trophic levels of food chains in terrestrial ecosystems. For instance, remarkably higher Cd concentrations in the viscera of herbivores and predators than the concentrations in their diets were observed in a Cd-contaminated semi-natural grassland [12]. Approximately 3% of Cd in soil can be transferred to human bodies via the consumption of rice grown in Cd-contaminated farmlands [13].

Parasites are defined as a group of organisms drawing nourishment from a host with only harmful but not immediate lethal effects on the host [14]. Some higher plants have evolved from autotrophic to hemiparasitic or even holoparasitic species. The former (also called ‘facultative parasite’) is still capable of photosynthesis thus can survive independently of hosts; while the latter (also called ‘obligate parasite’) has lost photosynthetic function thus fully relying on parasitism to hosts [15]. Among various holoparasites, *Cuscuta* sp. (Convolvulaceae), a.k.a. ‘dodders’, is a genus of rootless, leafless and string-like stem-parasites that develop connections to the shoots of host plants [16], and are recognized as worldwide agricultural weeds [17]. Newly germinated *Cuscuta* seedlings grow upward and rotate in the air until touching a point for attachment. Once attached and coiled around the stems or leaves of hosts, a special structure called ‘haustorium’ starts to develop at the contacting point and produce searching hyphae penetrating host tissues [18]. When arriving to the vascular bundles of hosts, the terminal cells of hyphae differentiate and respectively connect to the phloem and xylem of hosts [18,19]. After the establishment of these connections, *Cuscuta* becomes a super sink that compete for water, minerals and photosynthetic assimilates against other sink organs of host plants [20]. The processes of host searching and haustorium induction involve a detection of light quality signaled from host plants [19]. So far, the biological and ecological research of *Cuscuta* mainly focus on their interactions with hosts in the aspects of host selection [21], evolution and development of haustorium [22,23], impacts on host growth [24,25], and exchanges of substances and signals [18,26,27]. Meanwhile, studies of heavy metal stress (especially Cd stress) on the growth of *Cuscuta* are still scarce [28], and most of the published works limited their scopes to the physiological responses and detoxification mechanisms in *Cuscuta* [20,29].

Compared with the path from host plants to animal herbivores, the path from host plants to parasitic plants has received much less attention in the research of trophic transfer and biomagnification of heavy metals [28]. Among various parasitic plants, *Cuscuta* is believed as an ideal model species for studying parasitic trophic transfer of heavy metals in terrestrial ecosystems, since the rootless feature can guarantee that all heavy metals in *Cuscuta* are transferred from hosts without any direct uptake from soil [21]. However, so far to our knowledge, there is no experiment undertaken to investigate the transfer and accumulation of heavy metals, especially Cd, between *Cuscuta* and its hosts grown in contaminated soil. To investigate the transfer pattern and to test the occurrence of biomagnification of Cd in host-*Cuscuta* parasitic systems, we conducted a controlled greenhouse experiment using *C. chinensis* (‘dodder’ for simplicity, hereafter) as the parasite and soybean (*Glycine max*) as the host grown in soil with a series of Cd amendment levels. We examined the concentration, accumulation and allocation of Cd in various components (i.e., roots, stems and leaves of soybean, as well as dodders) of this soybean-dodder parasitic system, and also evaluated the transfer efficiency of Cd within this system.

## 2. Results

### 2.1. Biomass

**Figure 1:**
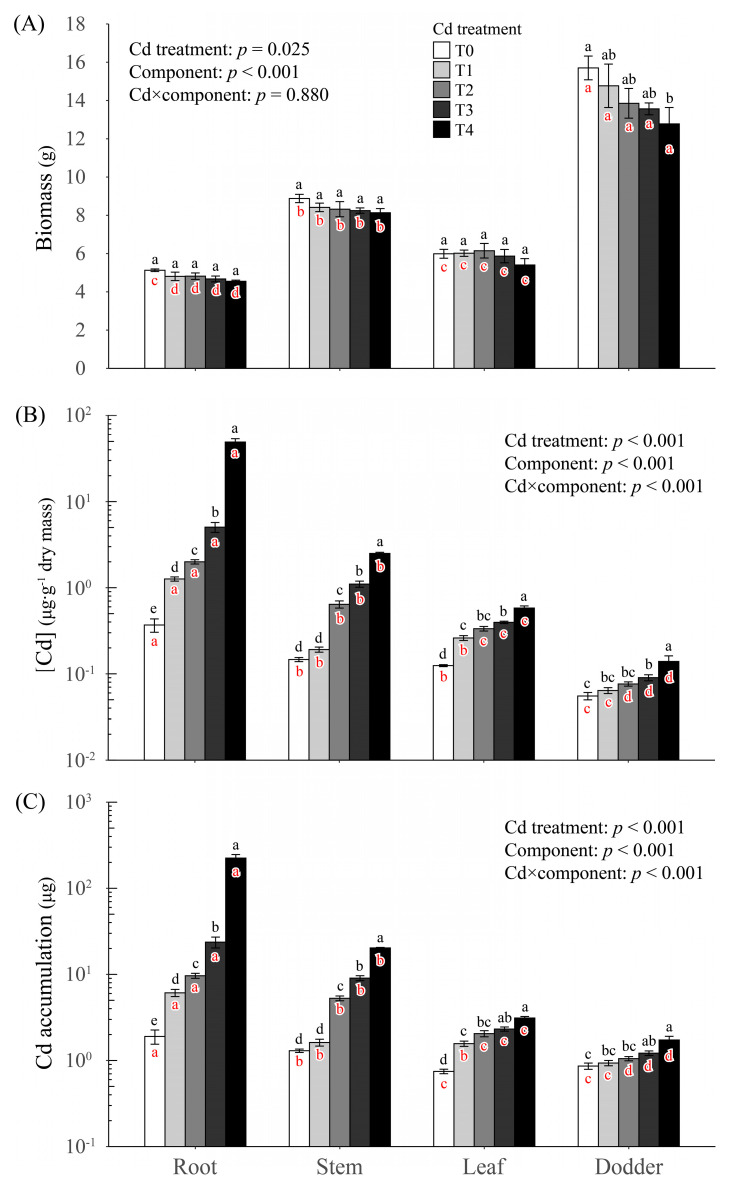
The effects of cadmium (Cd) treatment and component type on the (**A**) biomass, (**B**) Cd concentration ([Cd]) and (**C**) Cd accumulation of various components within the soybean-dodder parasitic systems. The levels of Cd treatment were applied as Hoagland solution (50% strength) amended with 0, 1, 10, 100, and 1000 mg·L^−1^ CdCl_2_, which are respectively marked as T0, T1, T2, T3, and T4. The analyses were performed using nested two-way ANOVAs with Cd treatment, component type and their interaction term as fixed factors, and pot replicate as a random factor, followed by Tukey’s post hoc tests. The results are presented here as *p* values of fixed factors, which were calculated based on type-III analysis-of-variance. Different black letters within one component type indicate significant differences between Cd treatment levels of that component. Different red letters within one Cd treatment level indicate significant differences between components under that treatment level. The error bars denote 1 SE of the mean.

**Figure 2:**
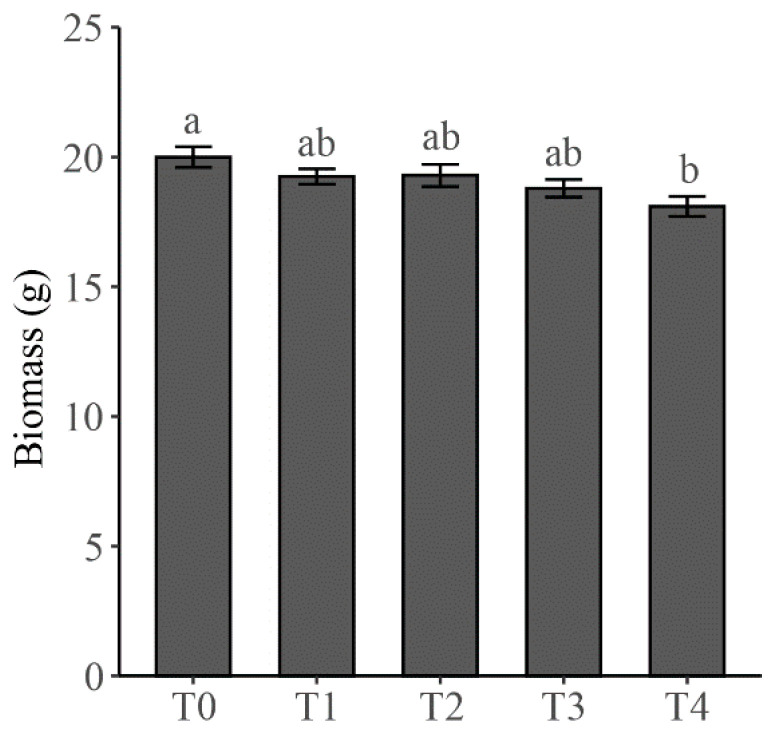
Total mass of soybean plants under different levels of cadmium (Cd) treatment. The levels of Cd treatment were applied as Hoagland solution (50% strength) amended with 0, 1, 10, 100, and 1000 mg·L^−1^ CdCl_2_, which are respectively marked as T0, T1, T2, T3, and T4. Different letters indicate significant differences between groups. The error bars denote 1 SE of the mean.

Cd treatment adversely affected the biomass of all components of the soybean-dodder parasitic system; however, significant reductions in biomass, as compared to that in T0, only occurred in dodders when the level of Cd treatment reached to T4 (Figure 1A). When we focused on the total mass of soybean (i.e., the sum of root, stem and leaf mass), it also tended to continuously decline with the increase of Cd treatment levels. Like the response of dodder mass, a significant reduction in total mass of soybean, as compared to that in T0, was only observed in T4 (Figure 2).

### 2.2. Cd Concentration

**Figure 3:**
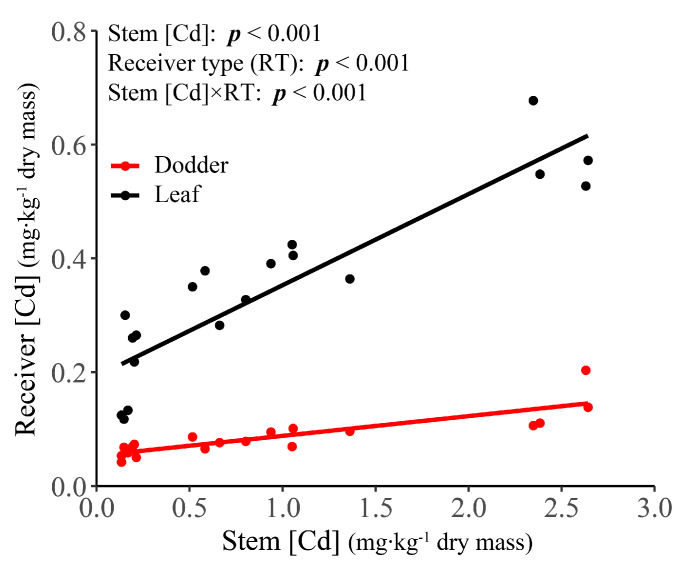
The correlations of cadmium concentration ([Cd]) between soybean stem (source) and its receiver (sink) components (i.e., soybean leaf and dodder). In the analysis (i.e., nested ANCOVA), stem [Cd] covariate, receiver component type and their interaction term were the fixed factors, and pot replicate was the random factor. The results are presented here as *p* values of fixed factors, which were calculated based on type-III analysis-of-variance.

With the increase of Cd treatment levels, Cd concentration ([Cd]) in all components also continuously increased. However, compared to [Cd] in T0, the first significant elevation was observed in T1 for roots and leaves, in T2 for stems, and in T3 for dodders (Figure 1B). Within this parasitic system, there was a general pattern that root [Cd] was always the highest, dodder [Cd] was always the lowest, and stem [Cd] and leaf [Cd] were always the intermediate (Figure 1B). However, the rank of [Cd] between stems and leaves appeared dose-dependent in response to Cd treatment. That is, stem [Cd] was similar as leaf [Cd] in T0 and T1 but became significantly higher than leaf [Cd] in higher treatment levels (Figure 1B). Both leaf [Cd] and dodder [Cd] were significantly positively correlated with stem [Cd] (t = 8.836, d.f. = 18, *p* < 0.001 for leaf; t = 7.029, d.f. = 18, *p* < 0.001 for dodder). However, for a given stem [Cd], leaf [Cd] was always higher than dodder [Cd] within the parasitic system; the extent of this difference enlarged with the increase of stem [Cd] (Figure 3).

### 2.3. Cd Transfer Coefficient

Transfer coefficient was defined as the ratio of concentration between sink and source components within the body of the same plant (or animal), or from different trophic levels. The transfer coefficient of Cd of various source-sink paths within soybean plants were almost always lower than 1 and generally declined with the increase of Cd treatment levels (Table 1). However, there were some exceptions. That is, the transfer coefficient of stem-leaf path in T1 was higher than 1 and was significantly higher but not lower than that in T0; and the coefficient of root-stem path in T1 was significantly lower but not higher than that in T2 and T3 (Table 1). Regardless of the levels of Cd treatment, Cd transfer coefficient of stem-leaf path was always significantly higher than that of root-stem path (Table 1).

When the transfer occurs between different trophic levels, a coefficient with value higher than 1 is a clear sign of biomagnification [30]. Clearly, the transfer coefficient of Cd of the stem-dodder path was always much lower than 1 and declined with the increase of Cd treatment levels without any exception (Table 1). In addition, Cd transfer coefficient of stem-dodder path was always significantly lower than that of stem-leaf path, regardless of Cd treatment levels (Table 1).

### 2.4. Cd Accumulation

The accumulation of Cd in a component was defined as the absolute amount of Cd in the component. The responses of Cd accumulation in the parasitic system were similar as the responses of [Cd] in the system. For all components, their Cd accumulations continuously increased with the increase of treatment levels. Compared to the accumulation of Cd in T0, the first significant increase was found in T1 for roots and leaves, in T2 for stems, and T3 for dodders (Figure 1C). The rank of Cd accumulation among components was root > stem = leaf > dodder in T0 and T1 but changed to root > stem > leaf > dodder in higher levels of Cd treatment (Figure 1C).

### 2.5. Allocations of Biomass and Cd

**Figure 4:**
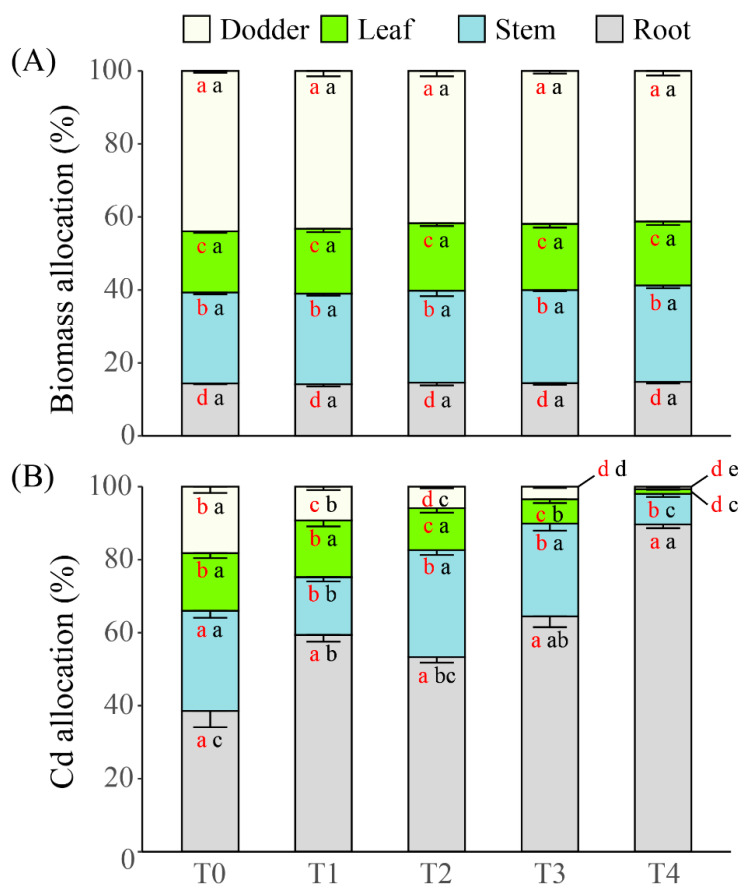
The allocation (i.e., proportional distribution) of (**A**) biomass and (**B**) cadmium (Cd) accumulation of various components within the soybean-dodder parasitic systems under different levels of Cd treatment. The levels of Cd treatment were applied as Hoagland solutions (50% strength) amended with 0, 1, 10, 100, and 1000 mg·L^−1^ CdCl_2_, which are respectively marked as T0, T1, T2, T3, and T4. Different black letters within one component type indicate significant differences between Cd treatment levels of that component. Different red letters within one Cd treatment level indicate significant differences between components under that treatment level. The error bars denote 1 SE of the mean.

Allocation here was defined as the biomass or Cd accumulation of a component in proportion to the total amount of biomass or Cd of the whole soybean-dodder system. Cd treatment had no effect on biomass allocation pattern of the parasitic system (F = 0.149, *p* = 0.963). Biomass of dodders always accounted for more than 40% biomass of the whole parasitic system; and the rank of biomass allocation among components was always: dodder > stem > leaf > root (Figure 4A).

Cd treatment significantly changed the allocation pattern of Cd accumulation within the parasitic system (F = 149.773, *p* < 0.001). With the increase of Cd treatment levels, Cd allocation to roots continuously increased (from ca. 40% to ca. 90%), while that to leaves and dodders continuously declined (from ca. 20% to ca. 1%). The responses of Cd allocation to stems were more complex. Compared to the allocation in T0, significant reductions were only found in the lowest (T1) and highest (T4) but not the intermediate levels (T2 and T3) of Cd amendments (Figure 4B). Without Cd amendment (i.e., in T0) to the parasitic system, the rank of Cd allocation was root = stem > leaf = dodder; however, along with the intensification of Cd amendment, the rank became root > stem > leaf > dodder (Figure 4B).

## 3. Discussion

By conducting a controlled greenhouse experiment, we examined the transfer, accumulation as well as allocation of Cd within a soybean-dodder parasitic system. Our findings of the limited Cd allocation in dodders accompanied with the Cd transfer coefficient of the stem-dodder path always being much lower than the value of 1, clearly demonstrated no sign of Cd biomagnification through the parasitic trophic transfer from soybeans to dodders, though both the concentration and accumulation (i.e., amount) of Cd in dodders did significantly increase with the levels of Cd treatment. Below, we discuss possible reasons that may explain such interesting findings.

The absence of Cd biomagnification in dodders may be attributed to a limited transfer of Cd from soybean plants. In line with the results of numerous studies (e.g., see reviews from [9]), the majority of Cd absorbed from soil was retained in the roots of soybean plants, the process of which is believed as a primary adaptive response to reduce Cd concentration thus moderating Cd toxicity to the aboveground of plants [9]. Due to insufficient discrimination of plants between Cd ions and other essential metal ions, Cd ions can be easily taken up by root cells from soil solutions [31]. However, once Cd ions entered root cells, most of them will be complexed (e.g., chelated) by a variety of organic ligands (e.g., phytochelatins, which belong to a family of peptides rich in cysteine and are synthesized from glutathione [3,32]). Subsequently, most of these Cd compounds will be either deposited and stored in the cell walls [33] or transported and sequestered in the intracellular organelles, the vacuole in particular [3]. By doing so, the concentration of free Cd ions can be largely reduced. However, a small proportion of Cd ions together with some Cd compounds will still diffuse towards xylem via plasmodesmata, and be transported to shoots via sap flow driven by transpiration [9]. During the transportation in stem, some of the Cd ions will be further complexed by ligands and fixed in the cell walls of xylem vessels [9]. This can further reduce the availability of soluble Cd to the sinks of stems, which were leaves and dodders in our case.

No occurrence of Cd biomagnification in dodders may be further attributed to their phloem feeder characteristics, as being a holoparasite [16]. Indeed, evidence from the research of heavy metal transfer through plant-invertebrate food chains suggests that phloem suckers are less likely to biomagnify Cd than chewers during their consumption of Cd-contaminated plants, due to the limited level of mobilized Cd in phloem saps of the plants [34,35]. However, to what extent the abovementioned two explanations can hold true remains questionable. For instance, compared to invertebrate suckers which almost only rely on phloem saps, the holoparasitic dodders also take up a great amount of saps from host xylem [19], which is the main route of Cd transfer from roots to shoots within the host. Such a bi-route feeding feature could put dodders in greater risks of higher doses of Cd uptake than invertebrate phloem suckers.

Therefore, this absence of trophic enrichment of Cd in dodders may imply that the uptake of substances, at least for some heavy metals, from soybeans to dodders was a selective rather than open process. Such an explanation sounds rather conflicting to the conclusions of quite a few works which suggested that the transfer from both xylem and phloem of hosts to dodders are non-selective, since substances ranging from micromolecules (e.g., minerals and photosynthates) to macromolecules (e.g., DNA and RNA) and even to pathogens (e.g., virus and phytoplasmas) were on the list [18,19]. Anatomical analyses also confirmed that during the formation of haustorium, dodders build open connections to both xylem [36] and phloem [23] of hosts. However, throughout the literature, we do find some supports to this selective uptake hypothesis. A field study from Boyd et al. [37] observed that *C. californica* accumulated higher concentrations of potassium and phosphorus but maintained a lower concentration of nickel (Ni) than its Ni-hyperaccumulator host *Streptanthus polygaloides*. Another one from Vurro et al. [20] also showed that when parasitizing wild carrot (*Daucus carota*) in a hydroponic condition, *C. campestris* had a lower level of [Cd], while a similar concentration of copper, but a higher concentration of zinc than the host.

Nevertheless, one can still argue that these findings can be attributed to the fact that toxic heavy metals (e.g., Cd and Ni), as compared to the essential elements, in the shoots of hosts are mostly in immobilized forms that cannot be taken up by dodders. However, there is still another piece of evidence in our study that can provide further supports to the selective uptake hypothesis. That is, both dodders and soybean leaves were the xylem sinks of soybean stems, thus should compete for the same solutes (including Cd ions) in the same stem xylem transferred from the same roots. Being a super-sink [20], dodders clearly overwhelmed this competition and took away most of the solutes, as indicated by a much higher level of biomass in dodders than in leaves. Then, we would expect a higher [Cd] or at least more accumulation of Cd in dodders than in leaves. In contrast, our results clearly showed an opposite pattern, and such a pattern continuously strengthened with the intensification of Cd exposure. Thus, the trophic transfer of Cd from soybeans to dodders appeared very likely to be a selective process (unfavored or less-selected in our case) and may also partially account for the absence of Cd biomagnification in our dodders.

Of course, we should not exclude the probability that no biomagnification in dodders may be the results of experimental setups. For example, the amendments of Cd to soil were given in the middle but not the beginning of experiments, so that the period (i.e., three weeks) of Cd treatment was not long enough to generate higher levels of [Cd] in dodders than in soybean stems. In addition, the efficiency of Cd transfer to some extent also depended on soil conditions [8]. For instance, soluble Cd ions are more available for plant uptake in acidic but not alkaline soils [9]; and the extent of immobilization of soil Cd is positively correlated with the level of organic matter in soils [38]. However, there is also evidence suggesting that in the presence of chloride plants tended to take up more Cd from soil thus facilitating subsequent Cd transfer [39]. As the amendment of Cd in our experiment was given in the form of CdCl_2_, and soybean roots had accumulated extremely high levels of Cd, the probability of our soil conditions being unsuitable for studying Cd trophic transfer is rather low.

Since Cd is extremely toxic to plants, an exposure to Cd, even at low concentrations, is expected to generate a series of detrimental effects on the growth of plants at both cellular levels (e.g., changing protein structures, reducing enzyme activities [40,41], inducing oxidative stresses [42]) and physiological levels (e.g., interrupting metabolisms [3], and interfering with water and mineral uptake [4]). Particularly for soybean, Cd exposure can significantly inhibit the photosynthetic rate by reducing chlorophyll content in leaves [43], and dampening nitrogen fixation activity by inducing nodule senescence in roots [44]. Furthermore, Cd exposure also can stimulate lignification of root cell walls, the process of which in turn can restrict the growth of roots in soybeans [45].

However, it was surprising that significant growth reductions of our soybeans only occurred in the highest level of Cd treatment. Such a ‘weak’ response might be since the growth of soybeans had already been strongly suppressed by dodders, the adverse effects of which largely masked the effects of Cd. Notably, to adequately test this ‘mask effect’ hypothesis, extra treatments of unparasitized soybeans should be included in the experimental design. The ‘weak’ response may also be attributed to the fact that soybeans were exposed to the Cd amendment after eight weeks of growth, by which time the plants have already passed the fast growth stage, thus yielding limited negative effects on the biomass accumulation. Indeed, most studies finding significant growth inhibitory effects had their plants treated with Cd at the seedling [43,45,46] or early growth stage [44]. Moreover, it also could be that the cultivar selected in our study happened to be a Cd-tolerant one. A growing body of literature demonstrated a genotype-dependent Cd tolerance in soybean, owning to the genotypic differences in e.g., the activities of enzymatic antioxidant system which is critical for the maintenance of membrane integrity thus redox homeostasis [47], the expressions of Cd-stress-response related MicroRNAs [48], and also the associations with arbuscular mycorrhizal fungi which play critical roles in alleviating Cd toxicity [49].

In addition to the biomass of soybean, the biomass of our dodder plants also appeared to be ‘weakly’ affected by Cd treatment. This could be attributed to a limited level of Cd transfer from soybean stems to dodders, so that [Cd] in dodders (except for that in T4) were still below the threshold of their body burden. In addition, similar as autotrophic plants, dodders also have evolved a series of physiological mechanisms, such as chelation and subcellular sequestration, to detoxify heavy metals [29]. For instance, the synthesis of phytochelatins plays key roles in chelating and sequestering Cd ions in plants [32]. In response to Cd exposure, dodders not only upregulate its own production of phytochelatins [20], but also take up a great amount of phytochelatins from host plants [29,50]. Such a response will strengthen their ability of Cd tolerance. Finally, as mentioned above, this ‘weak’ effect may also be attributed to the relatively short period of Cd exposure.

## 4. Materials & Methods

### 4.1. Plant Materials

*Cuscuta chinensis*, a.k.a. Chinese dodder, is an annual stem holoparasitic species characterized by rootless, leafless and string-shape yellow stems with a diameter around 1 mm. As a typical agricultural weed species, it is native to Asia and widespread in China and often parasitizes on plants of Fabaceae, Asteraceae, and Zygophyllaceae [51]. So far, the scientific community have limited their interests in the pharmaceutical values of *C. chinensis* [52,53], the biological and ecological significances of which have been overlooked until now, compared with other *Cuscuta* species, e.g., *C. australis* [27], *C. campestris* [20], *C. californica* [37], *C. japonica* [25], and *C. gronovii* [21]. A commercially available soybean (*Glycine max*) cultivar ‘white in August’, which is widely grown throughout China, was used as the host plants. Seeds of both Chinese dodder and soybean were obtained from local horticultural companies.

### 4.2. Experimental Design

This experiment was carried out in a semi-open greenhouse facility of Nanjing Forestry University from middle July (summer) to early October (autumn). Soybean seeds were surface-sterilized with a solution of 10% sodium hypochlorite for 5 min [54], and then thoroughly washed with distilled water and sown in moist sands. Three days later, germinated seedlings were transplanted into seedling trays for an initial growth of five days. Then, elder seedlings with healthy appearance and similar status were selected and transplanted to plastic pots (with a volume of 4 L) filled with commercial potting substrates (HAWITA, Germany) for experiment. The background level of Cd content in the potting substrates was around 0.133 mg∙kg^−1^ (dry weight) (see the determination method in Section 4.3). To promote the growth of soybean plants, they were regularly irrigated with 100 mL Hoagland solution (50% strength) twice a week. During the whole experiment, soybean plants were carefully watered daily in a manner that soils were kept moist but without water leakage from the bottom of pots.

The infection (or parasitism) of dodders started one week after the second transplanting of soybean plants, when the light environment under soybean shoots became suitable for the germination of dodder seeds, and the subsequent host searching and haustorium induction [19] of dodder seedlings (personal experience gained from a pilot study). Specifically, dodder seeds were immersed in concentrated (98%) sulfuric acid for 15 min to promote germination (i.e., to break seed dormancy by increasing the permeability of seed coat [55]), followed by thoroughly washing the seeds with distilled water. Then, the seeds were sown to soybean pots in a manner that each pot received 20 dodder seeds which were placed on soil surface and closely surrounded the stem of the soybean plant. Once the first successful attachment (or twining) of a dodder seedling on soybean stem was observed, the rest dodder seeds or seedlings that had not yet twined on the soybean stem were removed. This can guarantee that each soybean plant was successfully parasitized by one dodder plant (personal experience gained from a pilot study). Along with the growth of dodders, their adverse impacts on soybeans continuously intensified: the growth of soybean was visually arrested; the green leaves gradually turned yellow; the flowering was stopped, and the pods were no longer produced (even if produced, they were aborted at a very early stage) (personal observation). To prevent the death of soybean plants from the parasitism of dodders before the end of the experiment, the fertilization regime was adjusted to an irrigation of 200 mL Hoagland solution (50% strength) every two days from the sixth week after the second transplanting of soybean plants.

To avoid the overly inhibitory and toxic effects from high levels of Cd treatment on soybean plants at their early growth stages (which might greatly impede the infection and early development of dodder seedlings), the amendments of Cd were started eight weeks after the second transplanting of soybean plants, when soybeans had grown strong enough to withstand both dodder parasitism and high levels of Cd stress (personal experience gained from a pilot study). Plants were exposed to five levels of Cd treatment during the every-two-day fertilization events. That is, Hoagland solutions respectively amended with 0, 1, 10, 100, and 1000 mg·L^−1^ CdCl_2_ were given to the corresponding pots. These five levels of Cd treatment were respectively marked as T0, T1, T2, T3, and T4. In total, there were 20 pots with 20 dodder-parasitized soybean plants (i.e., four replicates per Cd treatment level). The treatment lasted for three weeks, because (i) there is evidence that significant Cd transfer from hosts to dodders can occur within two days after Cd amendment [20], and (ii) the time had just shifted from summer to autumn, gradually approaching to the end of growing season of the soybean cultivar in fields. During the three weeks, each dodder-parasitized soybean plant (i.e., soybean-dodder parasitic system) received 10 times of 200 mL CdCl_2_–contaminated Hoagland solutions in total. Notably, based on the fact that soil Cd contamination in China was in a range between 0.003 to 9.57 mg·kg^−1^ [56] and soil bulk density in China mainly distributed around 1.4 to 1.6 kg·L^−1^ [57], our rough calculations showed that such an extent of soil Cd contamination in China can be similar to 10 times irrigation of 200 mL solution amended with 0.01 to 50 mg·L^−1^·CdCl_2_ into a pot filled with 4 L soil (as used in our experiment). This range thus was well included in the chosen range of Cd treatment of our experiment. The higher levels (e.g., 100 and 1000 mg·L^−1^ CdCl_2_) of Cd treatment used here also enabled us to test whether the occurrence of Cd biomagnification in dodders is in a dose-dependent manner, e.g., biomagnification may only occur in high but not in low levels of soil Cd contamination.

### 4.3. Harvest and Measurements

Eleven weeks after the second transplanting of soybean plants (i.e., three weeks after the start of Cd treatment), the experiment was terminated, and the plants were harvested. Specifically, within the soybean-dodder parasitic systems, dodders were carefully separated from soybean plants. Subsequently, soybean plants were divided into roots and shoots. Roots were carefully washed free of soil, and shoots were further divided into biological stems, petioles and laminas. Regarding two reasons: (i) we found that dodders only had attached and formed haustoria into stems and petioles but not laminas of soybean plants, and (ii) both stems and petioles carried the function of resource transportation in soybeans, we pooled stems and petioles together and re-categorized them as ‘stem’ in the measurements and analyses. Laminas, which function as the sink receiving underground resources from stems, were also renamed as ‘leaf’ in the measurements and analyses. Then, all components of the soybean-dodder parasitic system (i.e., roots, stems and leaves of soybean, as well as dodders) were oven-dried at the temperature of 65 °C for three days.

The dry components were weighed, then grounded into powders and sieved through a 0.15 mm mesh for the measurements of [Cd]. Based on the test method from China National Food Safety Standard [58], [Cd] was determined with an inductively coupled plasma mass spectroscopy (iCAP RQ, Thermofisher, Waltham, MA USA) after nitric acid—hydrogen peroxide—hydrofluoric acid digestion. In addition, the background soil [Cd] in the potting substrates was previously determined. Based on China National Environmental Quality Standard for Soils [59], soil [Cd] was determined with an inductively coupled plasma atomic emission spectroscopy (iCAP 6300, Thermofisher, USA) after hydrochloric acid—nitric acid—hydrofluoric acid—perchloric acid digestion.

### 4.4. Statistical Analyses

Based on the biomass and [Cd] of various components, Cd accumulation ([Cd] × mass), and the allocations of biomass and Cd of the components were obtained. The transfer coefficient of Cd in different paths (i.e., root to stem, stem to leaf, and stem to dodder) were also calculated as the [Cd] ratio between sink and source components.

The effects of Cd treatment and component type on the biomass, [Cd], Cd accumulation, biomass allocation, and Cd allocation of various components within the soybean-dodder parasitic system, as well as on the Cd transfer coefficient of various paths within the system were examined using nested two-way ANOVAs with Cd treatment, component (or path) type and their interaction term as the fixed factors, and pot replicate as a random factor, followed by Tukey’s post hoc tests. The effects of Cd treatment on the total mass of soybean plants were also examined using a nested one-way ANOVA with Cd treatment as the fixed factor and pot replicate as the random factor, followed by Tukey’s post hoc test. In addition, the effect of receiver (i.e., sink) component type (i.e., leaf or dodder) on the correlation between stem [Cd] and its receiver [Cd] was also examined using a nested ANCOVA with stem [Cd] covariate, receiver component type and their interaction term as the fixed factors, and pot replicate as the random factor. All the statistical tests were conducted using packages ‘car’ [60], ‘lme4’ [61], ‘lmerTest’ [62], ‘LMERConvenienceFunctions’ [63], ‘emmeans’ [64], and ‘multcomp’ [65] in R v4.1.0 [66].

## 5. Conclusions

The current work is among the first to investigate Cd transfer from host plants to parasitic plants. We showed that among all components of the soybean-dodder parasitic system, dodders accounted for more than 40% biomass of the system but had the lowest Cd concentration and accumulated the least amount of Cd. Transfer coefficient of Cd between soybean stems and dodders was much lower than 1 and was also significantly lower than that between soybean stems and soybean leaves. These results suggested that the parasitism of stem holoparasite *C. chinensis* on Cd-contaminated hosts did not lead to Cd biomagnification. This may imply that the transfer of Cd from hosts to dodders was likely a selective process. This opinion deserves more tests since it could shed light on a new mechanism of heavy metal tolerance in parasitic plants.

## Figures and Tables

**Table 1:** Cadmium (Cd) transfer coefficients of various paths within the soybean-dodder parasitic system under different levels of Cd treatment.

Cd Treatment	Root-Stem	Stem-Leaf	Stem-Dodder
T0	0.44^a^_b_ (0.08)	0.85^b^_a_ (0.04)	0.38^a^_b_ (0.03)
T1	0.15^cd^_c_ (0.01)	1.40^a^_a_ (0.19)	0.34^ab^_b_ (0.04)
T2	0.32^ab^_b_ (0.03)	0.54^c^_a_ (0.07)	0.12^b^_c_ (0.02)
T3	0.23^bc^_b_ (0.02)	0.37^cd^_a_ (0.03)	0.08^c^_c_ (0.01)
T4	0.05^d^_b_ (0.01)	0.23^d^_a_ (0.02)	0.06^c^_b_ (0.01)

The levels of Cd treatment were applied as Hoagland solution (50% strength) amended with 0, 1, 10, 100, and 1000 mg·L^−1^ CdCl_2_, which are respectively marked as T0, T1, T2, T3, and T4. Different superscript black letters within one column indicate significant differences between different Cd treatment levels of the same path, while different subscript red letters within one row denote significant differences between different paths under the same Cd treatment level. Values in brackets denote 1 SE of the mean.

## Data Availability

The data presented in this study are available in the article. Additional data are available on request from the corresponding author.
